# High Homocysteine Levels During Pregnancy and Its Association With Placenta-Mediated Complications: A Scoping Review

**DOI:** 10.7759/cureus.35244

**Published:** 2023-02-20

**Authors:** Priyanka Thakur, Anuja Bhalerao

**Affiliations:** 1 Obstetrics and Gynaecology, N.K.P. Salve Institute of Medical Sciences & Research Centre and Lata Mangeshkar Hospital, Nagpur, IND

**Keywords:** recurrent pregnancy loss, placental abruption, pre-eclampsia, fetal growth restriction, placenta mediated complications, hyperhomocysteinemia, serum homocysteine

## Abstract

There is already abundant corroboration indicating that elevated serum homocysteine levels may be related to the risk of coronary, cerebral, and peripheral arterial diseases. High homocysteine levels have often been associated with placental vasculopathies and complications related to the placenta, such as fetal growth restriction, Abruption, hypertensive disorders of pregnancy, and recurrent abortions. This scoping review aims to integrate the currently available scientific literature and fill the gaps in our understanding of homocysteine metabolism during pregnancy and its relationship to placenta-mediated complications. Moreover, to summarize the existing literature on the correlation between raised maternal homocysteine levels in early gestation and its association with placenta-mediated complications. We developed this scoping review article by performing a literature review as per the Preferred Reporting Items for Systematic reviews and Meta-Analyses Extension for Scoping Reviews (PRISMA-ScR) guidelines and the search was conducted using PRISMA-S (an extension to PRISMA focusing on reporting the search components of systematic reviews) guidelines. The research question was clarified and modified using keywords with important literature published online between 2010 and 2022, which were included from PubMed, and Google Scholar databases with recognized titles and abstracts were examined and cross-checked for case overlap to choose the original reports. A summary of the descriptive data was organized according to the clinical manifestations (symptoms, imaging, and laboratory results) and outcomes (maternal and perinatal). In conclusion, a review of research papers from 2010 to 2022 showed that homocysteine levels during pregnancy fluctuate and are probably influenced by a population's regional, cultural, and socioeconomic status. According to the data, there is an association between elevated homocysteine levels and complications of pregnancies, such as early spontaneous abortions, pre-eclampsia, fetal development restriction, and abruption, as well as in certain cases of maternal and fetal mortality.

## Introduction and background

The problem or issue involves preventing maternal morbidity and mortality and improving fetal outcomes in patients with placenta-mediated complications by early detection of complications, such as fetal growth restriction, preeclampsia, abruption, and recurrent pregnancy losses. What is already known is that increased maternal homocysteine levels have often been associated with placenta and complications such as fetal growth restriction abruption, preeclampsia, recurrent pregnancy losses, and preterm birth.

Additionally, this paper adds that this scoping review provides a more precise knowledge of evidence that whether or not raised maternal homocysteine levels in early pregnancy are associated with placenta-mediated complications such as fetal growth restriction, hypertensive disorders of pregnancy, Abruptio Placentae, and recurrent pregnancy losses. Moreover, raised maternal serum homocysteine levels can be used as an early predictor of placenta-mediated complications.

There is already abundant evidence indicating that elevated serum homocysteine levels may be related to the risk of coronary, cerebral, and peripheral arterial diseases [1-3]. High homocysteine levels have also been associated with placental vasculopathies and complications related to the placenta, such as fetal growth restriction, abruption, hypertensive disorders of pregnancy, and recurrent abortions. There is a lack of knowledge on how hyperhomocysteinemia increases the likelihood of complications during pregnancy and other negative effects. According to a theory, high homocysteine levels lead to endothelial dysfunction, making women with the condition more likely to have it in the placental vasculature [2]. With the foregoing background, we aim to integrate the currently available scientific literature in order to fill gaps in our understanding of homocysteine metabolism during pregnancy and its relationship to placenta-mediated complications. Also, we aim to summarize the existing literature on the correlation between raised maternal homocysteine levels in early gestation and its association with placenta-mediated complications.

During gestation, the woman’s body prepares itself for the hemostatic challenge of delivery by creating a physiologically hypercoagulable state. However, this entails a higher risk of venous thrombosis and problems caused by the placenta, which poses significant difficulties for the mother and fetus. A significant problem for women's health is the prevention of these placenta-mediated problems, which altogether complicate up to 15% of pregnancies [1]. In developing nations, pregnancy-related problems affect 15% of women. Placenta-mediated problems, which account for 5-15% of all pregnancy-related complications, have considerable negative effects on both mother and the fetus. One of the Millennium Development Goals is to decrease the morbidity and mortality of mothers and children [2]. Fetal growth restriction, hypertensive disorders of pregnancy, placental abruption, and recurrent pregnancy loss are major sequelae of impaired placental function throughout pregnancy [3]. The relationship between placenta-mediated pregnancy complications (PMCs) and many indicators such as antiphospholipid antibodies (APLA), blood Vitamin B12 levels, and folic acid levels has recently been examined; however, the findings are still debatable and contradictory. Folate, Vitamin B12, and B6 are an integral part of homocysteine metabolism, hence it can prove to be a potentially more reliable marker of placenta-related problems. Moreover, recent studies have suggested that various pregnancy-related problems may be related to maternal plasma homocysteine levels.

Homocysteine, a sulfur-containing essential amino acid, is produced mostly by the demethylation of dietary methionine, which is necessary for the development of cells and tissues in the human body [4]. Homocysteine levels decline throughout typical human pregnancy. The main causes of this decline are a physiological rise in glomerular filtration rate during pregnancy, the rise in plasma volume and related hemodilution, and a hypothesized higher absorption of homocysteine by the fetus. Low circulating levels of folic acid and low dietary folate intake are also contributing factors as they are required for homocysteine metabolism. There is evidence suggesting that folic acid deficiency leads to an increased risk of preterm birth, low birth weight, fetal growth restriction, and neural tube abnormalities. Increased plasma total homocysteine levels are a metabolic consequence of folate insufficiency. Uncertainty exists as to whether a higher total homocysteine concentration is only a reflection of folate status or whether it is hazardous on its own due to vascular action [5]. Since the vascular alterations brought on by homocysteine are comparable to those brought on by hypertensive disorders of pregnancy, it can be assumed that high levels of homocysteine are linked to the hypertensive disorder spectrum. However, how hyperhomocysteinemia increases the likelihood of problems during pregnancy and other negative effects is unknown. According to a theory, high homocysteine levels lead to endothelial dysfunction, making women more likely to have it in the placental vascular system. The process through which elevated hazards are brought about by hyperhomocysteinemia is that methionine undergoes transmethylation, which produces homocysteine. Three enzymes, as well as several cofactor vitamins, are the main components of their metabolism. Hyperhomocysteinemia (HHcy) results from genetic abnormalities in these enzymes or a lack of these vitamins. The average biological definition of HHcy is a fasting value >15 mol/l. Only 3% of homocysteine in the circulation are disease-related, 75% are protein-bound (mainly to albumin), and the remaining 22% are in the disulfide form. Plasma concentrations of 5-15 mol/l were considered normal [6]. A change in a woman's homocysteine levels results in placental vasculopathies. Hypertensive disorders of pregnancy, abruption, fetal growth restriction, and recurrent miscarriages, all have a shared placental pathophysiology and are associated with aberrant placental vasculature. Thus, high levels of homocysteine directly affect the placenta making it more vulnerable to causing pregnancy complications that directly or indirectly affect both mother and fetus.

Among various studies, there is a lack of consistency in the reported results that support the link between maternal homocysteine concentrations assessed throughout each of the three trimesters of pregnancy and difficulties caused by the placenta [5]. Many studies have shown a significant association between hyperhomocysteinemia and placental diseases, and few have not shown any significant association. Therefore, more studies need to be conducted on the association between elevated blood homocysteine levels and placental complications in pregnant women in order to reach a conclusion [7]. Therefore, the goal of our study is to determine whether greater levels of maternal plasma homocysteine during early pregnancy are related to an increased risk of placenta-mediated problems. moreover, to assess its contribution in predicting the incidence of different placenta-mediated complications. The objective of this scoping review article is intended to identify knowledge gaps in various related studies and concise these lacunae in order to formulate a better understanding. Moreover, to apply this understanding in determining precisely whether an elevated maternal plasma homocysteine concentration in early gestation is linked to a higher risk of complications caused by the placenta or not.

## Review

Methods

Our primary research question is whether there is any association between raised maternal homocysteine levels in antenatal women in early gestation and placenta-mediated complications. Moreover, if there is any difference in the obstetric outcome in antenatal women with raised levels of serum homocysteine as compared to normal levels of homocysteine throughout gestation.

The studies included in this article were cross-sectional studies indicating subject randomization and controls in the group, prospective cohort and case-control studies, systematic reviews, and meta-analyses. The articles excluded were retrospective and non-controlled studies with no randomization practices. The population, Concept, and Context (PCC) format is described in Table 1.

**Table 1:** PPC Format: Inclusion and Exclusion Criteria PPC Format: The population, Concept, and Context Format

PCC format	Inclusion criteria	Exclusion criteria
Population	Cross-sectional studies -Prospective cohort studies -Case-control studies -Systemic reviews Meta-analysis -Singleton pregnancy	-Retrospective studies Non-controlled studies with no randomization -Diagnosed congenital anomalies in foetus -Known case of Chronic hypertension, renal disease, Diabetes Mellitus. - Other known causes of Recurrent Pregnancy Loss like Incompetent os, fibroid, and congenital anomalies of the uterus.
Concept	Knowledge and studies are available on hyperhomocysteinemia and its role in placenta-mediated complications.	
Context	Any healthcare hospital with a Tertiary healthcare center, any country	Primary or secondary health care system
Publication dates	All studies published in 2010 and onwards	Studies published prior to 2010
Language	Only English	Other than English studies

Our search strategy is based on a study format set by Population Concept Context (PCC), the research question. Throughout the process, the question was amended and clarified. Key papers were discovered by an automated search of the PubMed and Google Scholar databases using keywords, free words, and headings for medical subjects. Weekly updates and database adaptations were made to the search. In the additional material, a comprehensive search method for the PubMed database is provided as an illustration of one search. 

All quantitative, qualitative, and mixed-method research designs that were solely published in English were selected for the scoping review. Cross-sectional, exploratory, observational studies, time series, case-control studies, and cohort study designs were all taken into consideration. Studies using mixed approaches that matched the inclusion requirements were also examined. To now, a 2010-2022 date range has been specified as the maximum to get the most recent research on the topic.

To formulate this scoping review, relevant literature was reviewed and developed using Preferred Reporting Items for Systematic reviews and Meta-Analyses Extension for Scoping Reviews (PRISMA-ScR) guidelines, and the search was conducted using PRISMA-S (an extension to PRISMA focusing on reporting the search components of systematic reviews) guidelines. The research question was clarified and modified using keywords with important literature published online between 2010 and 2022, which were included from PubMed, and Google Scholar databases with recognized titles and abstracts were examined and cross-checked for case overlap to choose the original reports. A summary of the descriptive data was organized according to the clinical manifestations (symptoms, imaging, and laboratory results) and outcomes (maternal and perinatal).

The population, concept, and context (PCC) format was used for this (Table 1). Participants were pregnant women between 10 to 14 weeks of gestation who were carrying a singleton and seeking prenatal care at an ANC clinic or expecting to give birth at a tertiary care facility. The comprehensive knowledge and authentic studies available on hyperhomocysteinemia and placenta-mediated complications in antenatal women. The review considered scholarly papers from any country and any hospital with a tertiary healthcare setting. 

In our study, we considered study designs such as cross-sectional, exploratory, observational, time series, case-control studies, and cohort studies that were published only in the English language. Also, mixed methods studies that met the inclusion criteria were reviewed. We included studies only with a date of publication from 2010-2022 till date to retrieve the most recent literature present on the subject matter.

The research question is based on the Population Concept Context (PCC) format. We clarified and revised during the entire process using keywords and medical terminologies, a computerized search was done in PubMed and Google Scholar databases and key articles were found. The search was adapted across databases and updated every week. 

One hundred and forty-nine relevant articles were retrieved from database searches. These were reviewed and twenty-eight duplicates were removed. Two independent reviewers screened articles by titles and abstracts and any disagreements were settled with a third reviewer. Eighteen full-text articles were read, out of which 10 were selected in which raised maternal homocysteine level and its association with placenta-mediated complications such as hypertensive disorders of pregnancy, Fetal growth restriction, abruption, and recurrent abortions were taken into consideration. Eight full-text papers were removed, and hypertensive disorders of pregnancy such as pre-eclampsia, fetal growth restriction, placental abruption, and pregnancy losses were explained. These papers were disqualified since they made use of alternative terminology. 

**Figure 1:**
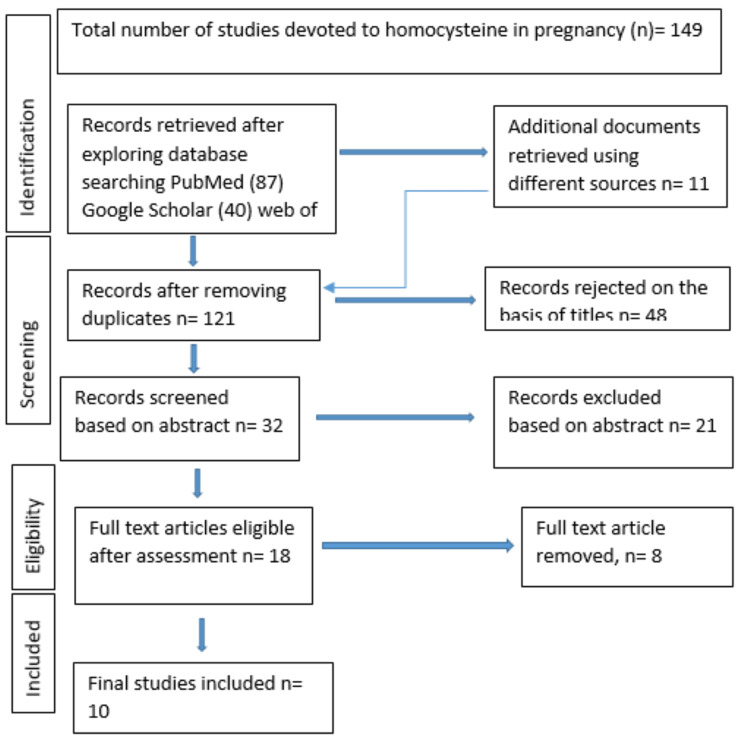
Prisma Flow Diagram

Data extraction was performed using extraction tools. Using this tool, we extracted specific data related to the study population, concept, and context, considering the details of the authors in the study, their country of origin, study design, instrument used for data collection, study objective, participant characteristics, and summary of the findings (Table 2). All articles are charted in the table and included in the final review (Table 2). A meta-synthesis approach was used to identify similarities and lacunae across studies. A scoping review aims to describe what is known about a subject and identify the loopholes and study gaps; thus, relevant portions that addressed the goal of the research were extracted and concepts were identified.

**Table 2:** Overview of the published studies in the review article

Sr no.	Study ID, year & country	Study design	Aim	Participants	Summary of findings
1	Bibi et al. 2010 [8]	Cohort study	To clarify the connection between blood homocysteine (hcy) levels and vascular-related pregnancy problems in expectant women and to evaluate the impact of folate, vitamin B12, and B6 on decreasing homocysteine levels.	A total of 332 prenatal women were enrolled, of whom 112 had normal, healthy pregnancies; 61 had pre-eclampsia; 49 had eclampsia; and 110 experienced placental abruption	Higher hcy levels have been seen in pregnancies affected by eclampsia and preeclampsia. The theory that folate, vitamin 812, and vitamin B6 reduce hcy levels in hyperhomocysteinemic women is supported by data.
2	Acılmıs et al. 2011 [9]	case-control study	The study intended to assess the connection between preeclampsia severity and to assess maternal and foetal blood levels of homocysteine, vitamin B12, folic acid, and homocysteine level in placental tissue	Pregnant women in 26 cases with mild preeclampsia, 26 cases with severe pre-eclampsia, and 26 cases of normal pregnancies.	Serum homocysteine levels in the mother and foetus were considerably raised in the severe pre-eclampsia group in contrast with the mild pre-eclampsia and control groups, indicating that elevated serum homocysteine levels may be related to the severity of pre-eclampsia. On the contrary, it appears that a lack of folic acid and vitamin B12 was not linked to increased blood homocysteine levels.
3	Mascarenhas et al. 2014 [10]		to review the connection of homocysteine levels in the first trimester (8–12 weeks) and foetal and mother outcomes.	In this cohort study, 100 pregnant women between the ages of 8 and 12 weeks participated.	Raised first trimester Serum homocysteine has been linked to preterm birth, hypertensive disorders of pregnancy, and a history of miscarriages. Pregnancy's low birth weight, oligohydramnios, meconium-stained amniotic fluid, and hypertensive disorders of pregnancy are also linked to this.
4	Wadhwani et al. 2015 [11]	Prospective cohort study	The study compares Hypertensive cases and normotensive control (NC) women in terms of their levels of maternal plasma folate, vitamin B12, and homocysteine from the commencement of pregnancy until delivery.	In the study, there were 62 instances of pre-eclampsia and 126 controls with normal blood pressure.	According to the study's findings, pregnant women with PE had elevated homocysteine levels throughout their whole pregnancy.
5	Maru et al. 2016 [12]	A comparative study	to establish the association between blood homocysteine levels, relevant laboratory tests, and pregnancy-related hypertension disorders.	This prospective study was placed over a period of two years. A total of 214 cases were investigated. They were split into four groups: those with eclampsia (32), moderate preeclampsia (64), and severe preeclampsia (50). And 68 controls.	The statistical bond between homocysteine values and the ferocity of hypertension and complications from hypertensive disorders is clear. It can be used as a genuine prediction marker for hypertension and the broad syndrome that it causes.
6	Mukhopadhyay et al. 2017 [13]	Prospective analysis	To ascertain the relationship between Hyperhomocysteinemia (HHCY) and recurrent miscarriages as well as the effects of folic acid (vitamin B 9), vitamin B 12, and HHCY on lowering its levels in the body and preventing obstetric problems.	A prospective research was conducted, and the blood homocysteine levels of pregnant women who had been admitted to our hospital over a two-year period and had a history of unexplained RPL were measured.	RPL and Hyperhomocysteinemia are related. Homocysteine levels are lowered when those with Hyperhomocysteinemia take vitamin supplements.
7	Gaiday et al. 2018 [14]	Systemic review	The goal of the study was to integrate the many pieces of currently available scientific evidence and fill in any knowledge gaps about Homocysteine levels in pregnancy and its relationship with certain pregnancy problems.	1287 research on Hcy and pregnancy complications looked at various effects and issues that might harm the mother and the foetus.	In review it was concluded that Homocysteine levels vary in an uncomplicated pregnancy. Then, throughout the second and third trimesters, the Hcy level usually decreases. A correlation between polymorphism and abortion has been shown in several research. There is enough evidence to support the link between HHcy and PE. High Hcy levels increased the likelihood of placental abruption by 5.3 times. However, the findings do not support the theory that Hcy levels are correlated with placental abruption.
8	Chaudhry et al. 2020 [5]	Cohort study	The purpose of this study was to find out if a higher level of maternal plasma homocysteine in the early to mid-second trimester is linked to a higher risk of placenta issues.	The cohort study includes 7587 individuals.	Their findings imply that placenta-mediated pregnancy complications are affected independently by early to mid-second trimester maternal homocysteine elevation.
9	Chamotra et al. 2020 [15]	Prospective cohort study	To ascertain the relation between excessively raised values of homocysteine during the antenatal period and unfavourable pregnancy outcomes.	180 pregnant women participated in the research, 57 of whom were exposed and 123 of whom were not.	The study provided the required proof that unusually high homocysteine levels are linked to pregnancy-related hypertension disorders and unfavourable pregnancy outcomes. Strongly conceived investigations and experiments are also required to better study the phenomena. Furthermore, it underlines the need for a quick and easy action that can help anticipate and avoid negative perinatal outcomes: assessing the much-ignored homocysteine levels throughout pregnancy.
10	Oluwole et al. 2020 [16]	Prospective cohort study	The study's goal was to find out how high maternal homocysteine levels affected the outcomes of pregnant Nigerian women in Lagos.	200 participants were taken in the study and divided into 2 groups.	In Lagos, there was a comparatively low rate of Hyperhomocysteinemia among mothers. Future prevention of these negative consequences may be affected by the associations between Hyperhomocysteinemia and unfavourable pregnancy outcomes.

Discussion

Homocysteine levels fluctuate during pregnancy, although they remain much lower than those in non-pregnant women. Since homocysteine levels tend to drop throughout pregnancy, some researchers have shown that the second trimester may be used to record a lower level. Although they decline in the second trimester and begin to rise thereafter, statistics suggest a normal gestation course. According to a study by Mascarenhas et al., from the eighth week of gestation to the mid-second trimester, homocysteine levels may steadily decrease over the course of pregnancy. Prior to eight weeks of pregnancy, the HYC level rises, and it also rises during the third trimester. Patients who experienced losses in the second and third trimesters, as well as those with hypertension, had significantly elevated homocysteine levels. In patients with elevated homocysteine levels, fetal growth restriction, oligohydramnios, meconium-stained liquor, and low birth weight babies are substantially more common. However, his findings were not significant for the correlation of elevated levels of homocysteine with preterm birth, gestational diabetes mellitus, high BMI, or pregnant women with fetal malformation [10].

According to Acilmis (2011), patients with severe preeclampsia had higher levels of homocysteine in their maternal and fetal plasma than patients with moderate preeclampsia and normotensive women [9]. According to reports, a high level of homocysteine in the blood is a risk factor for vascular disorders and endothelial dysfunction, which can result in preeclampsia [3]. Maru et al. concluded that homocysteine levels and pregnancy-associated hypertension are directly connected [12]. Atherosclerosis and endothelial dysfunction are two vascular alterations caused by hyperhomocysteinemia that are comparable to those brought on by preeclampsia and are brought on by a direct toxic and oxidative stress mechanism. Homocysteine levels and the severity and complications of preeclampsia and eclampsia were inextricably linked. Increased homocysteine levels have also been linked to maternal problems, such as abortion, retinopathy, multi-system organ dysfunction, and maternal death. Therefore, an extremely reliable predictive marker is needed to prevent placenta-mediated complications. Serum homocysteine testing is quick and simple and can be regarded as a reliable indicator of pregnancy-induced hypertension; however, larger-scale research is required before this test can be recommended [12].

Gaiday et al. had limitations because they did not consider literature written in languages other than English, French, German, and Russian, and they did not subject the collected data to a meta-analysis that might have provided additional information on the levels of homocysteine in pregnancy. The studies were not segmented according to demographic or geographic characteristics [14].

In their research, Chamotra et al. discovered that the exposed group's mean homocysteine level was substantially greater than that of the unexposed group. Additionally, neonates delivered to moms with hyperhomocysteinemia had a higher prevalence of low APGAR scores and NICU admissions [15]. Although further research is needed to prove this theory, inherited hyperhomocysteinemia has been hypothesized to be one of the causes of frequent miscarriages [17]. There is abundant data suggesting that raised homocysteine level is directly or indirectly connected to placental vasculopathies and its sequelae, but certainly, there are missing bits and pieces in this literature, which need to be assessed and assembled in order to establish a conclusion.

## Conclusions

A review of research papers from 2010 to 2022 showed that homocysteine levels during pregnancy fluctuate and are probably influenced by a population's regional, cultural, and socioeconomic features. According to the data, there is a connection between raised homocysteine levels and pregnancy issues such as early spontaneous abortions, preeclampsia, fetal development restriction, and abruption, as well as in certain cases of maternal and newborn death. Following the procedural folate metabolism, homocysteine is formulated from the *MTHFR* gene mutation. Further research is necessary to identify the genetic components underlying abnormalities in homocysteine metabolism. Further research needs to be done to precisely determine the timing during pregnancy when the investigation should be done (in our study it is 10-14 weeks of gestation) in order to conduct a preventative intervention in time and to prepare for the pregnancy-related complications that might follow. In summary, it is necessary to identify timelines for when testing should be made available and the cut-off values for each anticipated problem.
